# Bio-inspired self-shaping ceramics

**DOI:** 10.1038/ncomms13912

**Published:** 2016-12-23

**Authors:** Fabio L. Bargardi, Hortense Le Ferrand, Rafael Libanori, André R. Studart

**Affiliations:** 1 Complex Materials, Department of Materials, ETH Zurich, CH-8093 Zurich, Switzerland

## Abstract

Shaping ceramics into complex and intricate geometries using cost-effective processes is desirable in many applications but still remains an open challenge. Inspired by plant seed dispersal units that self-fold on differential swelling, we demonstrate that self-shaping can be implemented in ceramics by programming the material's microstructure to undergo local anisotropic shrinkage during heat treatment. Such microstructural design is achieved by magnetically aligning functionalized ceramic platelets in a liquid ceramic suspension, subsequently consolidated through an established enzyme-catalysed reaction. By fabricating alumina compacts exhibiting bio-inspired bilayer architectures, we achieve deliberate control over shape change during the sintering step. Bending, twisting or combinations of these two basic movements can be successfully programmed to obtain a myriad of complex shapes. The simplicity and the universality of such a bottom-up shaping method makes it attractive for applications that would benefit from low-waste ceramic fabrication, temperature-resistant interlocking structures or unusual geometries not accessible using conventional top–down manufacturing.

## Abstract (web summary)

Shaping ceramics into complex forms is a formidable goal. Here, the authors present an approach to self-shaping ceramics, inspired by self-folding processes in plants, in which the ceramic microstructure is embedded with aligned platelets that control the orientation of heat-induced shrinkage.

Ceramic parts with complex shapes find use in a multitude of applications, ranging from moulds used in the fabrication of gas turbine rotor blades to wire guides in the textile manufacturing, to microgears for micro-electromechanical systems^1^,^2^. Processing of ceramic components exhibiting intricate geometries generally involves top–down fabrication technologies such as injection molding^3^, *in situ* coagulation or gelation^4^,^5^,^6^, machining of pressed powder compacts^7^,^8^ or additive manufacturing techniques^9^. The resulting shaped parts are typically dried and subjected to a sintering process that enables densification of the particle compact. Although these processing methods lead to a wide range of geometries, complex and intricate ceramic pieces such as hinges, chains or encapsulated ceramics can only be achieved by co-firing cut and glued green bodies, which leads to weak interfaces at the junctions^10^. Thus, developing alternative processing techniques to fabricate advanced ceramics exhibiting intricate and complex shapes, while retaining good integrity and robust mechanical properties, still remains an open challenge.

Given that complex geometries are usually defined during the shaping step, a common requirement in all ceramic manufacturing routes is that no shape distortion takes place during drying and sintering. Warpage, for example, is often an issue in the manufacturing of multilayered ceramics^11^. Indeed, gradients in particle distribution or heterogeneous drying rates can lead to severe internal stresses, cracking and unwanted distortions of the piece^12^. Similar detrimental effects are also expected during sintering of the consolidated part when non-uniform packing density and/or unequal heat diffusion rates are present^13^. Such distortions are ultimately caused by inhomogeneous shrinkage in different regions of the same part during the drying and/or sintering processes.

Despite being considered problematic for many industrial processes, differential swelling/shrinkage is a strategy often used in plant systems to cause deliberate shape change on an external stimulus^14^. Typically, the swelling/shrinking degree of a hemicellulose soft matrix is restricted in specific directions through the preferential alignment of cellulose fibrils. Applying this design strategy in man-made composites has proven to be remarkably efficient for the fabrication of smart polymer-based materials whose shape change is triggered by variations of environmental conditions^15^,^16^,^17^.

Here we present a simple method to fabricate ceramic parts that self-shape into intricate geometries during the sintering process. Inspired by the organization of reinforcing cellulose fibrils in the plant kingdom, self-shaping is achieved by designing microstructures in which alumina reinforcing platelets are deliberately aligned to control the local shrinkage of a surrounding matrix of alumina nanoparticles (Al_2_O_3_ NP) during densification. In contrast to machining processes and injection molding routes currently used to produce complex-shaped ceramics, this bio-inspired approach minimizes the amount of waste material and does not require costly machinery. In addition, the method allows us to fabricate ceramic parts with complex geometries that do not exhibit weak interfaces or junctions. The bottom-up strategy outlined here is also versatile and can potentially be adapted to a wide range of materials for the fabrication of glasses or ceramics with additional functionalities such as transparency or piezoelectricity. As opposed to an elegant recent approach based on compressive buckling stresses arising from the deposition of metals, polymers or combinations thereof on a pre-strained substrate^18^, the present approach is indeed based solely on the development of internal strains that can be remotely programmed within the microstructure of the ceramic green body to generate shape changes in much larger structures up to the centimetre scale.

## Results

### Processing strategy

**Figure 1:**
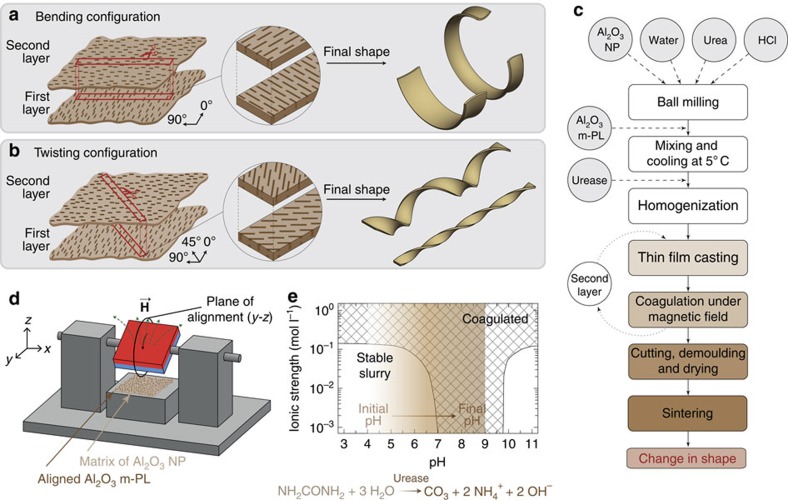
Design and processing of microstructured bilayers programmed to self-shape during sintering. Bilayer samples containing platelets orthogonally aligned in each layer can result in (**a**) bending or (**b**) twisting, depending on the microstructure within the strip. (**c**) Flowchart of the procedure developed to prepare programmable self-shaping ceramics. (**d**) Schematic representation of the setup used for platelet alignment. Biaxial alignment occurs along the plane in which the magnetic field is rotating, which corresponds to the (*y*–*z*) plane in our coordinates. (**e**) Colloidal stability diagram showing the pH change induced through the catalysed decomposition of urea by the enzyme urease, which was used to fix the microstructure in each layer after magnetic alignment. This graph was plotted using data from Gauckler *et al*.^20^

To demonstrate our ability to create self-shaping ceramics, we design proof-of-concept bilayer architectures whose microstructure is programmed to promote pure bending or pure twisting during sintering (Fig. 1). The microstructure required to induce differential shrinkage and lead to a predictable shape of the final sintered ceramic is introduced in the green body through biaxial alignment of alumina micrometre-sized platelets in the initially fluid cast suspension. Such platelet orientation control restricts the shrinkage of the continuous ceramic matrix along the alignment axes when subjected to sintering. Microstructures required to yield bending and twisting shapes are presented in Fig. 1a,b. The flowchart of the overall process to achieve those microstructural configurations is schematically shown in Fig. 1c. Magnetized alumina platelets (Al_2_O_3_ m-PL) are incorporated in a suspension of spherical Al_2_O_3_ NP and biaxially aligned using rotating magnetic fields (Fig. 1d)^19^. Magnetization is carried out by coating the microplatelets with 13% surface coverage of superparamagnetic iron oxide nanoparticles, following the protocol proposed by Erb *et al*.^19^ The magnetized Al_2_O_3_ m-PL are on average 10 μm long and 250 nm thick, whereas the Al_2_O_3_ NP exhibit an average diameter of 180 nm. After magnetic alignment, the suspension is consolidated through an enzymatically-triggered coagulation process to fix the oriented platelets in place^20^.

In a typical formulation, 50 vol% of Al_2_O_3_ NP are first dispersed through ball-milling in an aqueous solution containing urea at pH 4. To prevent fracture and reduction in dimensions of Al_2_O_3_ m-PL during the milling process, these particles are incorporated in the slurry only later by manual mixing. Additional water is also added to maintain the total solid contents at 50 vol%. After cooling the resulting slurry to 5 °C, the enzyme urease is carefully mixed into the suspension. Once the mixture is brought to room temperature, the enzymatic activity is enhanced and urea is decomposed into ammonia, according to the chemical reaction (equation (1)):


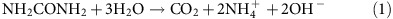


The increase in pH promoted by the enzymatic decomposition of urea leads to the destabilization of the slurry, following a process known as direct coagulation casting^20^, whose principle is illustrated in Fig. 1e. When the pH of the slurry approaches the isoelectric point (IEP) of the Al_2_O_3_ NP, the suspension coagulates and fixes the position and orientation of the magnetized Al_2_O_3_ m-PL.

### Coagulation kinetics and aligned microstructures

The degree of platelet alignment is maximized by carefully controlling the initial viscosity of the slurry and tuning the coagulation kinetics of the suspension. Although a very low initial viscosity is expected to speed up the alignment dynamics, it might also lead to undesired platelet sedimentation. Thus, the suspension viscosity should be high enough to minimize sedimentation, but at the same time sufficiently low to enable magnetic alignment of platelets. We observe that fluids exhibiting viscosities of 10 Pa.s at a shear stress of 0.01 Pa are already too viscous to enable quick alignment, reducing the maximum fraction of aligned Al_2_O_3_ m-PL to only 75% (see Supplementary Fig. 1, Supplementary Table 1 and Supplementary Methods for more details). Therefore, we choose this value of viscosity as the upper limit for achieving satisfactory platelet alignment.

**Figure 2:**
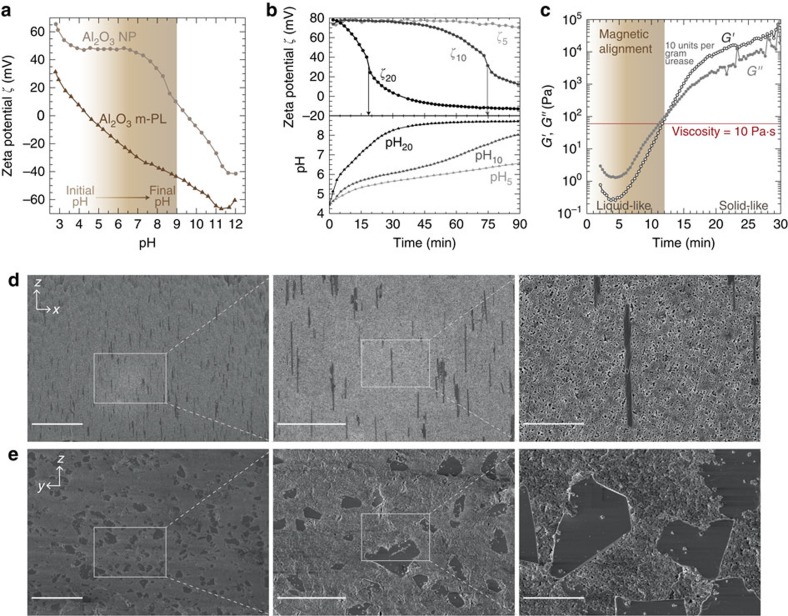
Consolidation kinetics and microstructured ceramics. (**a**) Determination of the zeta potential of the Al_2_O_3_ NP and the Al_2_O_3_ m-PL used in this study. (**b**) Evolution of the zeta potential (ζ_*x*_) and pH (pH_*x*_) during coagulation of the slurry depending on the urease concentration in enzymatic units, where *x* refers to the enzyme concentration in units of urease for 1 g of alumina. (**c**) Storage (*G'*) and loss (*G''*) moduli as a function of time for a suspension containing 50 vol% of particles at a Al_2_O_3_ NP:Al_2_O_3_ m-PL volume ratio of 90:10, 0.5 wt% of urea with respect to alumina and 10 units per g of total alumina of urease. (**d**,**e**) Scanning electron micrographs at different magnifications of polished cross-sections of green bodies cut along (**d**) the (*x*–*z*) plane and (**e**) the (*z*–*y*) plane (scale bars from left to right, 50, 20 and 5 μm).

Precise control over the kinetics of the slurry's coagulation is a prerequisite to successfully apply the direct coagulation casting method for shaping ceramic-based materials. As outlined in Fig. 1e, coagulation occurs by a homogenous *in situ* increase of the pH such that the initial positive electrostatic charges on the particle's surface are neutralized, leading to agglomeration through attractive van der Waals interactions. In Fig. 2, we show the zeta potential of the Al_2_O_3_ particles present in the ceramic slurries used throughout this study and how the enzyme-catalysed reaction is used to manipulate the colloidal stability and rheology of the suspension. In the absence of organic dispersants and at pHs lower than the IEP of 9, a high positive absolute zeta potential is measured for the Al_2_O_3_ NP suspended in water, confirming the possibility of achieving colloidal stability through a purely electrostatic mechanism. As the Al_2_O_3_ m-PL are labelled with superparamagnetic iron oxide nanoparticles, their IEP is shifted to a low value close to pH 4, as shown in Fig. 2a (see Supplementary Fig. 2 and Supplementary Methods for further details on the zeta potential analysis). Al_2_O_3_ m-PL are present only in a small fraction of 10 vol% of the suspended alumina content. Owing to their different IEPs, we expect such platelets to be covered by Al_2_O_3_ NP in the pH range from 5 to 9 and, therefore, behave similar to the other oppositely charged Al_2_O_3_ NP present in the slurry.

Once the conditions required for initial colloidal stabilization are established, one can control the speed of the enzymatic reaction and thus the kinetics of the coagulation process, by adjusting the amount of urease added to the suspension. The enzymatic activity of the urease is expressed in units per gram of pure urease, where one unit corresponds to the amount of urease necessary to release 1 μmol of reaction product per minute at 24 °C in water^6^. We define here the concentration of urease as the number of enzymatic units per gram of alumina in the slurry. The dependence of the coagulation kinetics on the enzyme concentration was evaluated by measuring the time evolution of the zeta potential of the particles, which should approach zero as the pH is shifted towards the IEP. For practical reasons, we define the coagulation time as the inflection point in the zeta potential curves, as highlighted by the arrows in Fig. 2b. Increasing the amount of urease from 5 units per gram of total alumina to 20 units per gram reduces the coagulation time from longer than 90 min to ∼18 min. These kinetic experiments show that using 10 units per gram of urease leads to an intermediate coagulation time of ∼75 min.

Although zeta potential data for different enzyme concentrations provide a first indication of the suspension coagulation kinetics, the relatively dilute conditions used in these experiments (1 vol% of total solid loading) might not be representative of the actual kinetics involved during magnetic alignment (50 vol%). Thus, the speed of coagulation was also assessed by investigating the time evolution of the rheological properties of a typical slurry containing 50 vol% alumina and 10 units of urease per gram of alumina. Figure 2c shows that the slurry undergoes the sol-gel transition (*G′′/G′*=1) within a time scale of ∼12 min. As expected, this time is much shorter than that determined by zeta potential analysis. The gelling point of this slurry occurs at a global dynamic viscosity of 10 Pa·s (at 0.5 Pa), which indicates that magnetic manipulation of platelets are unlikely to take place at longer times. In the present case, the time span of 12 min used for the magnetic assembly proved to be long enough to allow for platelet alignment with very low angle variability. For example, green bodies with a nominal alignment angle programmed at 90° exhibit platelets oriented at an average angle of 89.5°±3.57° after sintering, as depicted in Fig. 2d,e and Supplementary Fig. 3. Although agglomerates of about two to three platelets are observed after the magnetic assembly process, the spatial distribution of platelets within the continuous nanoparticle matrix is similar to those obtained in monomer solutions exhibiting much lower viscosity^21^. As in polymer-based composites obtained using such monomer solutions, the presence of small agglomerates should not cause any deleterious effect in the programmed shape change as long as they are fully oriented along the alignment plane. In the following, we focus on the shape changes that occur during the sintering of green bodies processed from slurries containing 10 vol% Al_2_O_3_ m-PL relative to the total solid content of 50 vol% and coagulated using 10 units per gram urease.

### Programming the macroscopic change in shape

**Figure 3:**
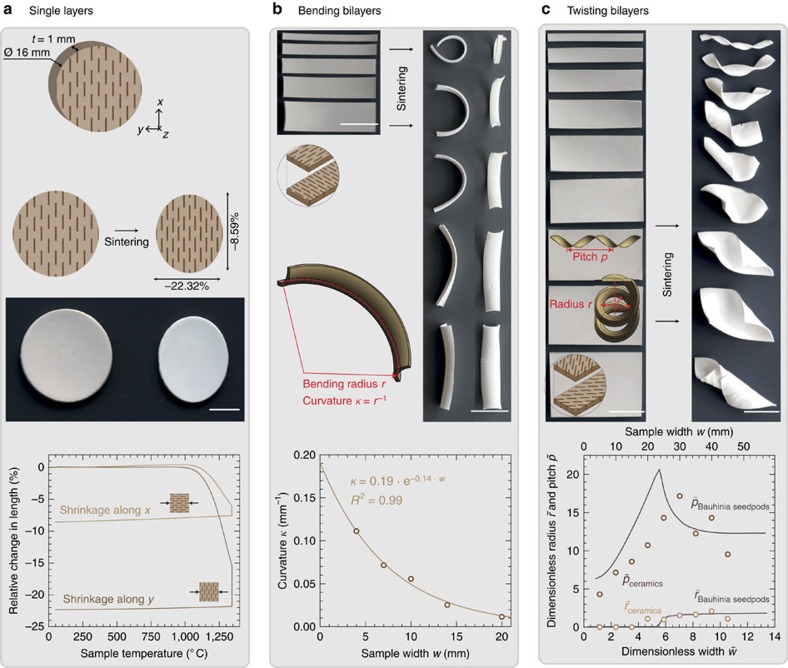
Macroscopic shape changes of ceramic bilayers of variable width. (**a**) Single layers containing aligned platelets and their anisotropic shrinkage upon sintering (scale bar, 5 mm). Total shrinkage perpendicular to the platelet alignment direction (*y*-direction) is about three times larger than that parallel to the platelet face (*x*-direction). (**b**) Sintering-induced bending of bilayers cut at 90° relative to the platelet orientation (*y*-direction; scale bars, 25 mm). The curvature in the long dimension decays exponentially with the sample width. (**c**) Sintering-induced twisting of bilayers cut at 45° with respect to the platelet orientation (scale bars, 25 mm). The dimensionless radius and pitch as a function of the width (symbols) follow a similar trend as observed for natural seedpods (full lines)^23^.

To produce self-shaping ceramics that bend or twist during sintering, bilayer strips are prepared by sequential casting. First, a 1 mm-thick layer of the slurry is spread and placed under a rotating magnetic field to align the Al_2_O_3_ m-PL in the *y*–*z* plane according to the setup shown in Fig. 1d. After complete coagulation, a second layer of the suspension with the same thickness is spread on top of the first one followed by alignment of the Al_2_O_3_ m-PL in the plane perpendicular to the first layer (*x*–*z* plane in Fig. 1d). Next, the coagulated bilayers are divided into strips at well-defined angles of 90° or 45° relative to the direction of alignment of the first layer (Fig. 1a,b). The cut strips are then dried to form mechanically stable green bodies that are eventually sintered at 1,600 °C for 1 h. This cutting step is a way to generate both bending and twisting shapes from the same bilayer film. Alternatively, cutting can be avoided by casting the suspensions directly in a mould of the intended geometry and changing the direction of the mold relative to the magnet. Strips extracted at an angle of 90° with respect to the direction of alignment of the platelets in the first layer undergo pure bending deformation, whereas twisting is observed for strips that are cut at an angle of 45° relative to the *y*-direction (Fig. 3b,c).

Changes in shape occur during sintering because of the orthogonal anisotropic shrinkage in each of the microstructured layers, which creates internal stresses that can only be reduced by undergoing deformation^13^,^22^,^23^. Figure 3a quantifies the sintering-induced anisotropic shrinkage in different directions for a single-layered alumina containing 10 vol% of Al_2_O_3_ m-PL aligned within the (*x*–*z*) plane. On sintering at 1,300 °C for 1 h, a total linear shrinkage of 8.6% is measured parallel to the platelet orientation (*x*-direction), whereas as much as 22.3% shrinkage is observed perpendicular to the platelet orientation (*y*-direction). Sintering at higher temperatures should lead to increased anisotropic shrinkage as the densification process is still incomplete at 1,300 °C. The strong dependence of the local shrinkage on platelet orientation occurs because the Al_2_O_3_ m-PL behave as hard inclusions within the continuous matrix of packed Al_2_O_3_ NP that undergo densification at lower sintering temperatures. In the conditions of the experiments described in this study, the sintering behaviour of the Al_2_O_3_ NP is the driving force for the shrinkage of the continuous matrix around the stiff microplatelets. The extent of shrinkage in each individual microstructured layer is eventually determined by the orientation of the microplatelets in that layer. In analogy to the biological counterpart, the reduction in surface area of particles during sintering plays the same role in reducing the total free energy of the system as the chemical hydration of hemicellulose molecules during swelling in plant seedpods.

We do not expect the formation of any liquid phase in a system containing only Al_2_O_3_ and Fe_3_O_4_, and sintered in air^24^. However, the substantial shrinkage of the nanoparticle matrix near the edges of the microplatelets, where the densification rate is expected to be higher, might lead to the formation of a dense alumina network as described by Ozer *et al*.^13^ The formation of this network is a possible reason for the incomplete densification of the final ceramic part, which reached a maximum relative density as high as 89.1±0.1 vol% after sintering at 1,550 °C, as indicated by the plateau observed in the graph of relative density as a function of sintering temperature (Supplementary Fig. 4).

The densification of the ceramic parts is achieved through conventional diffusion processes within the matrix nanoparticles during sintering. Remarkably, the density levels and grain sizes observed are typical for alumina samples processed under similar conditions^25^. Higher densities and smaller grain sizes can potentially be obtained by incorporating minor amounts of additives that are known to promote densification and inhibit grain growth during sintering^26^. Moreover, the use of nanoparticles with multimodal size distribution as the continuous matrix can potentially increase the final density of the ceramic piece through improvement of the packing density in the neighbouring areas around the larger platelets^27^.

The dependence of the shrinkage of the powder compact on the platelet orientation can be harnessed to programme the final shape of sintered parts based only on their initial geometry and microstructure. Such programmability requires the determination of well-defined correlations between the final shape (output parameter) and the initial geometry and microstructure (input parameters). To demonstrate this programmability, we established correlations between the final output shape and the geometrical input parameters for bending and twisting bilayers of variable relative widths (Fig. 3b,c and Supplementary Movie 1). For the pure bending system, the curvature, *κ*, was used to describe the final output shape. In twisting bilayers, the radius, *r*, and pitch, *p*, of the obtained helicoidal structures were taken as output parameters describing the final shape of the sintered parts.

The curvature along the longest dimension is found to decrease exponentially with increasing the width of the bending strips, resulting in a reference curvature *κ*_0_ of 0.19 mm^−1^ (Fig. 3b). The well-defined correlation obtained enables programming of the final curvature by simply changing the width of the bilayer strips. For the twisting system, the radius and the pitch of the ceramic bilayers were found to exhibit a similar qualitative dependence on the width as that previously observed for the natural seedpod *Bauhinia variegate*^23^. In general, the shape change of narrow and wide strips are dominated by the bending and stretching elastic energies of the system, respectively. To allow for a direct comparison of our results with those obtained for the seedpod, we normalize the measured absolute radius (*r*), pitch (*p*) and width (*w*), resulting in the dimensionless quantities: 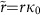, 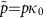 and 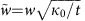, respectively. *κ*_0_ is the reference curvature determined in Fig. 3b, whereas *t* corresponds to the thickness of the bilayer. The observed dependence of 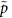 on 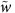 is characterized by a peak at a critical width of the bilayer strips, 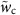. This critical width is found for intermediate widths at which bending and stretching energies are equally important and has been predicted from numerical simulations through an energy minimization procedure (Fig. 3c)^23^. The shift observed in the transition peak between the natural and the synthetic twisting strips might result from the additional curvatures observed along the shortest dimension of the samples. This creates a saddle-like shape in the bilayers, which is not observed in natural seedpods^28^.

The bilayered microstructure generated in the green parts is preserved in the final piece but the platelets slightly deviate from the angle of alignment during sintering due to the development of internal strains and the change in macroscopic shape (see Supplementary Fig. 5). To characterize the mechanical properties of the pieces after sintering we performed Vickers indentation by applying a load of 500 kgf on cross-sections of samples exhibiting various bending curvatures ranging between 0 and 0.12 mm^−1^. Polished surfaces were indented with the cross-section of the platelets facing the surface (similar as in Supplementary Fig. 5). The hardness values obtained for the sintered ceramic pieces were comparable for all tested samples with an average of 10.33±0.66 GPa. This value is comparable to those of commercial alumina with similar densities (∼3.6 g cm^−3^), which typically range from 10.4 to 11.8 GPa (https://www.coorstek.com/ and http://global.kyocera.com). Although fully densified ceramic parts were not achieved with the formulation used in this study, the implementation of strategies that maximize particle packing and green density should allow for a significant improvement on the mechanical performance of such bio-inspired self-shaping ceramic materials.

Thanks to an accurate control over the sample thickness throughout the study, the final pitch and curvature are very reproducible. Within a set of six samples, we measured an average thickness of 1.04±0.08 mm after sintering for 1 h at 1,600 °C. If the thickness is measured along seven different positions within one single sample a variation of only 0.05 mm (<5%) is obtained. In addition, we observed pitch variability of only 4.8% in three twisting samples exhibiting the same width and thickness but varying length (see Supplementary Fig. 6).

### Complex shaped ceramics

**Figure 4:**
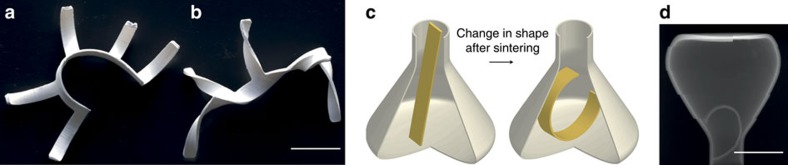
Programmed self-shaping as a method for designing complex ceramic pieces. Ceramic parts with complex shapes resulting from site-specific (**a**) bending or (**b**) twisting effects on sintering (scale bar, 20 mm). (**c**) Cartoon illustrating a ceramic piece with complex shape that cannot be easily engineered with conventional processing methods. In this simple example, a bilayered consolidated green body is programmed to bend during sintering and become trapped within the ceramic vessel. (**d**) Experimental realization of the effect shown in (**c**), as illustrated by a two-dimensional slice extracted from X-ray tomography of the object after sintering (scale bar, 20 mm).

As the final shape of the strips after sintering is solely controlled by their geometry and microstructure, even more complex objects with programmable self-shaping behaviour can be obtained. As illustrative examples, Fig. 4a,b demonstrate some of the intricate geometries that can be achieved through the combination of orthogonal bending motions or twisting effects in the same ceramic object. The initial geometry contains five short arms with a bilayer microstructural configuration that are connected by a longer bilayer exhibiting another microstructure. The local platelet alignment in each one of these different parts is locally programmed to result in distinct site-specific shape changes. The object shown in Fig. 4a is obtained by cutting the microstructured bilayers along one of the platelet alignment directions to achieve bending effects. Platelet alignment within the bilayers is programmed to result in bending of the five arms towards a given direction, whereas the connecting strip curves in the opposite direction during sintering. Using the same strategy, strips cut at an angle of 45° with respect to one direction of alignment result in complex geometries that combine multiple twisting effects (Fig. 4b). These examples illustrate how the site-specific microstructure of bilayers can be exploited together with the initial geometry of the green body to obtain a wide range of complex shapes in the final ceramic.

The processing strategy outlined here has the potential to produce stiff ceramic pieces that could not be fabricated without using post-processing techniques that very often impair the mechanical performance of the final piece. Figure 4c shows a ceramic element trapped within a vessel using the shape change induced by sintering (please refer to Supplementary Movie 2). In this case, a straight green body bilayer presenting a microstructural configuration programmed for bending is placed in a sintered alumina vessel with a narrow opening. After sintering at 1,600 °C for 1 h, the bilayer undergoes bending and gets trapped within the thermally stable ceramic vessel. The sintered ceramic bilayer can no longer be removed without fracturing either the vessel or the ceramic piece. The configuration of the bilayer within the vessel after sintering can be observed in the two-dimensional slice extracted from X-ray microtomography shown in Fig. 4d.

Similar to plant systems^22^, the broad design space that can be explored to affect the final curvature of the deformed samples, namely sample thickness, Young's modulus and shrinkage ratio, provides great flexibility to the method proposed here. However, it should be noted that the finite size of the reinforcing platelets limits the minimum thickness attainable for each layer to the largest dimension of the platelet inclusion. Smaller thicknesses would require the use of smaller inclusions and higher magnetic fields for particle orientation. Moreover, the range of complex shapes that can be generated with the system outlined here is restricted to geometries that are intrinsically prone to bending or twisting in at least one specific direction. The stripe or plate geometries shown in this and in previous reports are illustrative examples of the complex shapes that are accessible^16^,^23^.

Programmable self-shaping through anisotropic microstructural design is an elegant method to fabricate unique complex ceramic objects. Based on magnetic alignment of hard anisotropic micro-inclusions and the enzymatically triggered coagulation of ceramic slurries, microstructured bilayers of consolidated green bodies with bending and twisting deformation mechanisms that resembles those of plants and seedpods can be fabricated. Owing to its simplicity, the procedure described here has the potential to be expanded to other types of ceramic-based materials such as glasses and electric or magnetic ceramics, paving the way for the so far unexplored manufacturing of complex-shaped or high-temperature responsive stiff elements.

## Methods

### Magnetization of Al_2_O_3_ m-PL

Magnetized Al_2_O_3_ m-PL were prepared following a procedure published elsewhere^19^. In a typical experiment, 10 g of Al_2_O_3_ m-PL (Ronaflair, WhiteSapphire, 10 μm diameter, 250 nm in thickness) were introduced into 300 ml of deionized water and stirred magnetically. Ferrofluid EMG 705 (0.375 ml; Ferrotec, Germany) was pre-diluted in 200 ml of deionized water and added to the 300 ml suspension containing the platelets. The suspension was stirred at room temperature until the supernatant became clear. The platelets were then filtered and washed with 1,500 ml of deionized water and 250 ml of ethanol. Finally, the magnetized platelets were dried at 160 °C for 24 h.

### Preparation of the slurry

Alumina nanopowder (Al_2_O_3_ NP, TM-DAR, Taimei, Japan, high purity >99.99%, average particle size 180 nm, surface area 13.7 m^2^ g^−1^) was first dispersed in deionized water at 50% solid loading and maintained at pH 4.5 (HCl, >37%, puriss. p.a., Sigma-Aldrich, Germany). Subsequently, 0.5 wt.% of urea (puriss. p.a., Sigma-Aldrich) with respect to the alumina was added to the suspension. The slurry was de-agglomerated and mixed in a lined rolling mill with high-purity alumina balls of 10 mm diameter as grinding media for 24 h. Magnetized platelets were added to the suspension to achieve a volume ratio of 90% Al_2_O_3_ NP and 10% Al_2_O_3_ m-PL. Deionized water was added to adjust the total solid content to 50 vol%. The slurry was then cooled to 5 °C in a water–ice bath. A solution of urease (Type IX, 62,000 units per gram solid, Sigma-Aldrich) was prepared at a concentration of 1,000 units ml^−1^.

### Casting

The slurry was homogeneously spread on a Teflon substrate with dimensions 10 × 10 cm^2^ with a spatula and covered with aluminum foil, to prevent excessive water evaporation. A vertically rotating magnetic field of about 60 mT was applied for 1 h. We chose to apply the magnetic field for a longer time than the 12 min required, to ensure not only alignment but also sufficient coagulation of the suspensions to enable manipulation of the wet bodies in the next steps. For bilayers, the substrate was then turned by 90° with respect to the direction of the first alignment and a second layer was poured on top. After 1 h, the sample was cut into pieces with a sharp razor blade in the desired cutting angles and sizes. The pieces were peeled off carefully from the substrate and dried in air at room temperature for a minimum of 2 h.

### Sintering

The green bodies were sintered in an oven (Nabertherm, Switzerland) in a ceramic crucible filled with bare platelets to allow for the deformation of the piece. Samples were first heated at 1 °C min^−1^ up to 400 °C and held at 400 °C for 2 h, then heated at 1 °C min^−1^ up to the sintering temperature of 1,600 °C for 1 h before cooling to room temperature at 20 °C min^−1^.

### Suspension characterization

The zeta potential and pH measurements were conducted with an electro-acoustic spectrometer (DT-1200, Dispersion Technology Inc., USA). Aqueous suspensions of the different powders and mixtures (1 vol% solid loading) were ultrasonicated at 50% of the total power (UP200S, Dr Hielscher) for 5 min before the measurements and the initial pH was adjusted with HCl (1 N, Merck, Germany). Titration was performed by addition of a NaOH aqueous solution (1 N, Merck).

### Gelation kinetics

The dynamic viscosity of the slurry was determined with a stress-controlled rheometer (Gemini 200, Bohlin Instruments, England) in oscillation mode at 1 Hz. The measured viscosity was found to be constant up to an applied shear stress of 0.6 Pa. The gelation kinetics was determined by oscillatory measurements at 1 Hz at 0.5 Pa. All measurements were carried out using a vane tool to prevent wall slipping in these high solid-loading suspensions.

### Microstructure analysis

Cross-sections parallel and perpendicular to the direction of the platelet alignment were cut out from the green bodies. The fractured surface was then polished by broad ion beam (Hitachi IM4000, Japan) for 2.5 h (6 kV, deviation angle C3). Next, the samples were sputtered with 5 nm of platinum and investigated by scanning electron microscopy (LEO 1,530, Zeiss, Germany). The degree of alignment was quantitatively assessed using image analysis (Image J software) by extracting the alignment angle of 100 platelets within one image. The alignment angle distribution in a green body is fitted by a Gaussian function that is centered at 89.5° and presents a standard deviation of 3.58°. These values indicate that the magnetic assembly process leads to a very small variability in the alignment angle of platelets.

### Shrinkage ratio measurements

The linear shrinking ratio of microstructured single layers as a function of temperature along the *x*- and *y*-directions as described in Fig. 3a was determined using an optical dilatometer (DIL 806, TA Instruments, Waters, Switzerland) on disk-shaped samples of 15 mm diameter and 2 mm thickness.

### Hardness measurement

Samples were embedded in an epoxy resin (Specifix-20 kit resin, Struers, USA) and mechanically polished using a combination of polishing grits with particle sizes of 125, 46 and 18 μm. Vickers hardness was measured using a load of 500 kgf on a macro indenter (MXT-a, Wolpert, Germany).

### Macroscopic shape characterization

The shape change of the microstructured ceramic bilayers after sintering was captured by a regular camera. The dimensions and specific parameters for the bending/twisting movements were directly measured on the samples. For the bilayer piece trapped within the ceramic vessel, several X-rays scans of the samples were taken using a CT scanner (μCT100, Scanco Medical AG, Brüttisellen, Switzerland) with a field of view of 60.5 mm and at an X-ray voltage and current of 90 kVp and 200 μA, respectively.

### Data availability

The data that support the finding of this study are available from the corresponding author on request.

## Additional information

**How to cite this article:** Bargardi, F. L. *et al*. Bio-inspired self-shaping ceramics. *Nat. Commun.* **7,** 13912 doi: 10.1038/ncomms13912 (2016).

**Publisher's note:** Springer Nature remains neutral with regard to jurisdictional claims in published maps and institutional affiliations.

## Supplementary Material

Supplementary Information Supplementary Figures, Supplementary Table and Supplementary Methods.

Supplementary Movie 1 Shows the change in shape in a self-twisting microstructured green body bilayer during sintering.

Supplementary Movie 2 Summarizes the steps to trap a ceramic piece inside a ceramic vessel.
