# Supplementary material for: Bio-inspired self-shaping ceramics
Source: Nat Commun. 2016 Dec 23;7:13912. doi: 10.1038/ncomms13912 (PMC5196359; doi:10.1038/ncomms13912)
Supplement: Supplementary Information — Supplementary Figures, Supplementary Table and Supplementary Methods. [file ncomms13912-s1.pdf]

**Supplementary Figures**

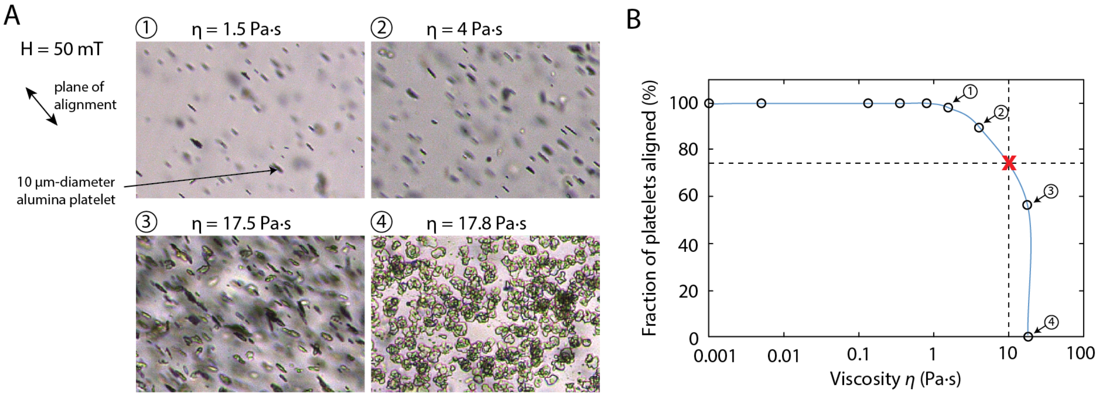


**Supplementary Figure 1|** Influence of the background viscosity on the alignment degree of alumina platelets under the effect of a 50 mT rotating magnetic field. **A)** Optical images and **B)** Fraction of platelets aligned perfectly in the direction of the magnetic field in function of the background viscosity.

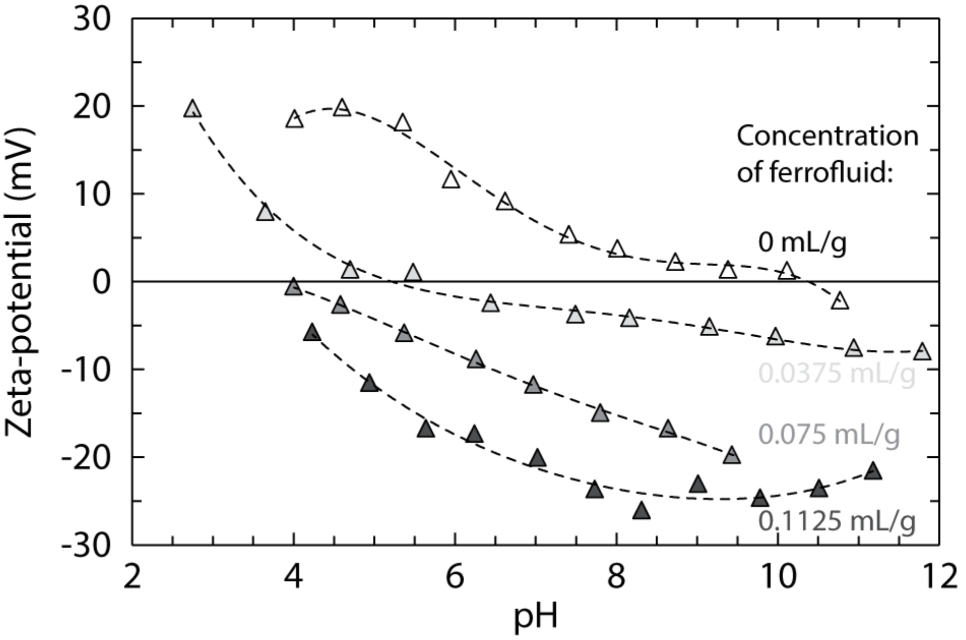


**Supplementary Figure 2|** Zeta-potential as a function of pH for alumina platelets modified with different amounts of ferrofluid, indicated as milliliter of ferrofluid per gram of platelets (mL/g).

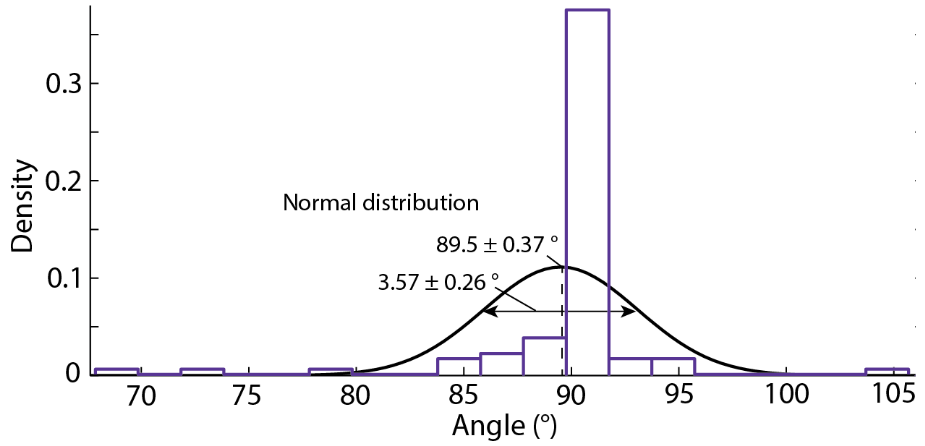


**Supplementary Figure 3|** Distribution of alignment angles within a cross-section of a sample containing vertically aligned platelets.

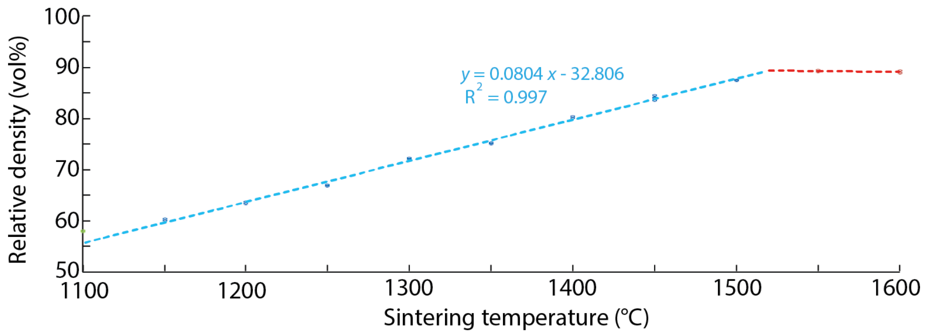


**Supplementary Figure 4|** Evolution of the relative density of a twisting ceramic as a function of the sintering temperature. The maximum density (89.1 ± 0.14 vol%) is achieved around 1550°C as shown by the plateau region indicated in red in the graph.

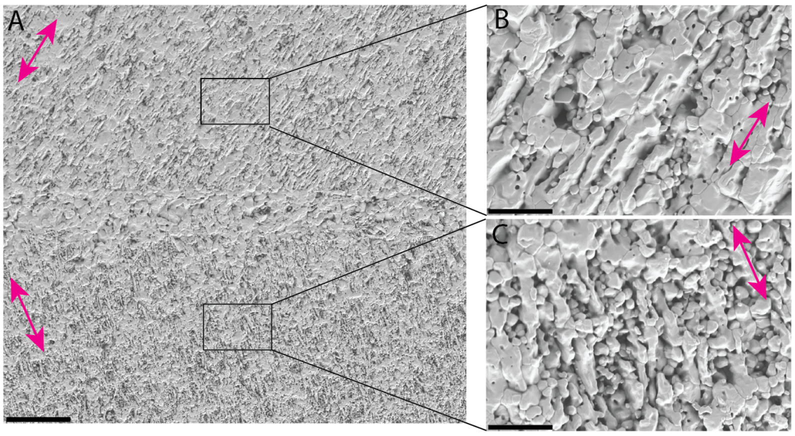


**Supplementary Figure 5|** Electron micrograph of a cross-section of a twisted ceramic after sintering at 1600°C for one hour showing that the bilayered

microstructure is kept even after undergoing significant macroscopic deformations (**A**, scale bar, 50 μm). Higher magnification images (**B,C**, scale bars, 10 μm) show the direction in which the platelets are aligned in each layer, as indicated by the arrows. The initial microstructure of the specimen before sintering exhibited vertically aligned platelets in both layers.

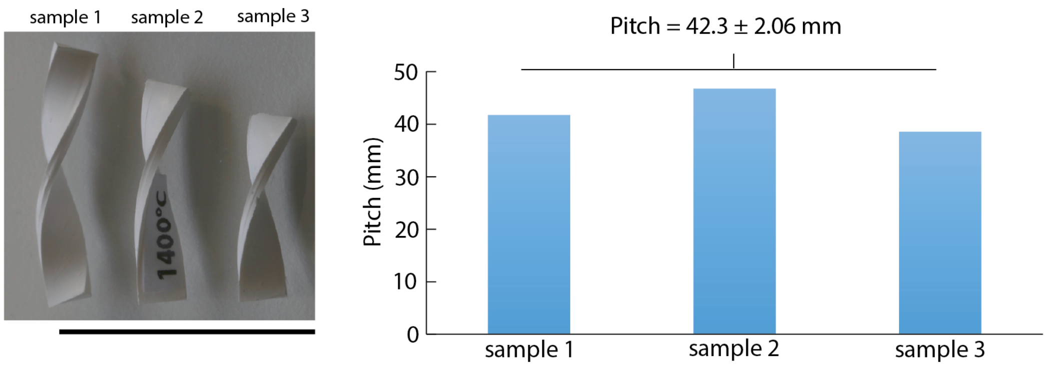


**Supplementary Figure 6|** Reproducibility of the pitch measured in three samples programmed to undergo twisting during sintering at 1400°C (scale bar, 25 mm).

**Supplementary Table**

**Supplementary Table 1|** Viscosities of the liquids used in the experiment. The results with the four liquids with the highest viscosities are presented in Supplementary Figure 1.

| Viscosity (Pa.s) | Liquid description | Number in Supplementary Figure 1 |
|---|---|---|
| 0.001 | Distilled water | |
| 0.05 | Corn oil (Sigma) | |
| 0.14 | 10 wt% PolyVinylPyrrolidone in water (MW 360 000, Sigma) | |
| 0.35 | Triton X-100 (Acros Organics) | |
| 0.8 | Standard oil (DKD, ZMK Sachsen-Anhalt | |
| 1.5 | 10 wt% Aradur (GY250, Hunstman, Belgium) and 90 wt% Araldite (CH917, Hunstman, Belgium) | (1) |
| 4 | 25 wt% Aradur and 75 wt% Araldite | (2) |
| 17.5 | 100 wt% Aradur with viscosity of 17.5 Pa.s | (3) |
| 17.8 | Carrageenan gel (Sigma, Germany) | (4) |

**Supplementary Methods**

**Determination of the maximum background viscosity allowing the magnetic alignment of the alumina platelets**

Under an optical microscope (Leica, Switzerland), dilute suspensions containing 0.1 - 1 vol% alumina platelets are prepared in a pill glass containing liquids with dynamic viscosities ranging from 0.001 Pa.s to 17.8 Pa.s and measuring according to the procedure described in the manuscript. The optical observation is carried out in the pill glass next to a permanent magnet (Neodymum, Supermagnete, Switzerland) rotating at a frequency of 4 Hz. The magnetic field strength at the position where the suspension is added was measured using a Gaussmeter (Lakeshore, USA) that indicated 50 mT. We used the liquids listed in Table 1. Image analysis reveals the maximum viscosity allowed that for reaching a high degree of platelet alignment which is defined here as fractions of aligned platelets on the plane of the rotating magnetic field higher than 75%. By data interpolation, we estimated the maximum viscosity to allow for high degree of platelet alignment to be around 10 Pa.s (Supplementary Figure 1).

**Modification of the zeta-potential of alumina micro-platelets with the magnetization**

The adsorption of superparamagnetic iron oxide nanoparticles (SPIONs) on the surface of bare alumina platelets is expected to affect the resulting net surface charge. Indeed, zeta potential measurements as a function of the pH revealed that the isoelectric point (IEP) is considerably shifted towards lower pH with increasing surface coverage of SPIONs (Supplementary Figure 2). Throughout this study, we fixed the amount of ferrofluid at a concentration of 0.0375 mL per gram of alumina platelet, which results in an IEP at a pH of about 5, whereas the bare platelets present an IEP at a pH of about 10.

**Procedure used to record the change in shape during sintering**

A green body stripe of 1 mm thickness and 25 mm length containing the twisting configuration was placed in an optical fleximeter (TA Instruments, Expert Solution Systems, Italy) on two holding rods of alumina separated by 20 mm. The sample is heated up to 1600 °C at a rate of 15 K/min and illuminated by a blue LED at 478 nm and recorded by a digital camera with a resolution of 0.5 μm per pixel of the camera.
